# microRNA-182 targets special AT-rich sequence-binding protein 2 to promote colorectal cancer proliferation and metastasis

**DOI:** 10.1186/1479-5876-12-109

**Published:** 2014-05-01

**Authors:** Min-Hui Yang, Jiang Yu, Dong-Mei Jiang, Wen-Lu Li, Shuang Wang, Yan-Qing Ding

**Affiliations:** 1 Department of Pathology, Nanfang Hospital, Southern Medical University, Guangzhou 510515, China; 2 Department of Pathology, School of Basic Medical Sciences, Southern Medical University, Guangzhou 510515, China; 3 Department of General Surgery, Nanfang Hospital, Southern Medical University, Guangzhou 510515, China

**Keywords:** miR-182, Colorectal cancer, SATB2, Metastasis, EMT

## Abstract

**Background:**

Increasing evidence has revealed that microRNAs (miRNA) played a pivotal role in regulating cancer cell proliferation and metastasis. The deregulation of miR-182 has been identified in colorectal cancer (CRC). However, the role and mechanism of miR-182 in CRC have not been completely understood yet.

**Methods:**

The expression levels of miR-182 in CRC tissues and CRC cell lines were examined by performing stem-loop quantitative RT-PCR. The stable over-expression miR-182 cell lines and control cell lines were constructed by lentivirus infection. Subsequently, CCK-8 assay, plate colony formation assay, cell migration, invasion assay and experimental animal models were performed to detect the biological functions of miR-182 *in vitro* and *in vivo*. A luciferase reporter assay was conducted to confirm target associations. Western blot and immunohistochemical analysis were performed to examine the expression changes of molecular markers that are regulated by miR-182.

**Results:**

We found that miR-182 expression is increased in CRC cells that originated from metastatic foci and human primary CRC tissues with lymph node metastases. The ectopic expression of miR-182 enhanced cell proliferation, invasion, and migration *in vitro*. Stable overexpression of miR-182 also facilitated tumor growth and metastasis *in vivo* too. Further research showed that miR-182 could directly target the 3’untranslated region (3’UTR) of SATB2 mRNA and subsequently repress both the mRNA and protein expressions of SATB2, which we identified in previous studies as a CRC metastasis-associated protein. Restoring SATB2 expression could reverse the effects of miR-182 on CRC cell proliferation and migration. Investigations of possible mechanisms underlying these behaviors induced by miR-182 revealed that miR-182 induced epithelial-mesenchymal transition (EMT) by modulating the expression of key cellular molecules in EMT.

**Conclusions:**

Our results illustrated that the up-regulation of miR-182 played a pivotal role in CRC tumorigenesis and metastasis, which suggesting a potential implication of miR-182 in the molecular therapy for CRC.

## Background

Colorectal cancer (CRC), a common digestive tract tumor, is considered as the third leading cause of cancer-related deaths all over the worldwide [1]. Although several kinds of treatments have been developed recently for the patients with CRC, poor prognosis continues to be in patients with advanced CRC [2]. Metastasis of CRC cells to vital organs is responsible for the majority of cancer deaths. Despite years of studies, the molecular underpinnings of CRC metastasis remain incompletely understood.

microRNAs (miRNAs) are a cluster of non-coding RNA molecules whose size ranges 19 to 24 nucleotides, and suppress target gene expression by initiating mRNA degradation and/or blocking translation [3]. miRNAs have diverse functions, including the regulation of cellular differentiation, proliferation, apoptosis, and carcinogenesis and metastasis [4-6]. Researches have found that miR-182 plays an important role in regulating mammalian circadian rhythm, T-cell development and DNA repair [7,8]. Several studies have illustrated that miR-182 is abnormally expressed in various tumors [9,10] and directly involves in human cancer processes, such tumorigenesis, migration and metastasis [11-15]. It has been identified that the expression of miR-182 is up-regulated in CRC [10,16,17]. This up-regulated expression of miR-182 can be correlated with the poor prognosis of patients with CRC [10]. However, the role and mechanism of miR-182 in CRC have not been completely understood till date. In this study, we explored the role of miR-182 in CRC and found that the up-regulation of miR-182 promoted the proliferation, invasion, and metastasis of tumor cell by suppressing SATB2 in CRC. In addition, we explored the underlying mechanism of miR-182 functions in CRC. Therefore, our data demonstrated that miR-182 was indeed a metastasis promoter in CRC, and the study also shed light on the molecular mechanisms of its anti-metastasis function in CRC patients.

## Methods

### Tissue specimens and cell culture

Fresh and formalin-fixed, paraffin-embedded, CRC specimens were obtained from patients who were diagnosed with primary CRC and then underwent elective surgery in Nanfang Hospital, Southern Medical University (Guangzhou, China). The tissues that have been used for conducting this study have been approved by the ethics committee of Nanfang Hospital, Southern Medical University. In total, 31 cases of fresh CRC tissues were freshly frozen in liquid nitrogen and stored at -80°C until further use.

The human embryonic kidney cells 293T and the human CRC cell lines DLD-1, HCT116, SW480, SW620, and Lovo were obtained from a cell bank at the Chinese Academy of Sciences (Shanghai, China). A subclone named M5 with enhanced metastatic abilities in liver and a subclone named SCP 51 with enhanced metastatic abilities in liver and lymph node were isolated by *in vivo* selection of SW480 cells through a process described in our previous studies [18-20]. All CRC cell lines were cultured in RPMI 1640 medium (Gibco, Gaithersburg, MD, USA) with 10% fetal bovine serum (HyClone, Logan, USA) and 100 U/ml penicillin/streptomycin (Gibco). They were maintained in a humidified chamber with 5% CO_2_ at 37°C. 293T cells were maintained in Dulbecco’s modified Eagle’s medium (DMEM) that was supplemented with 10% FBS.

### RNA isolation and quantitative real-time PCR

Total RNA was extracted using TRIzol Reagent (Invitrogen, Carlsbad, CA). cDNA was synthesized using the PrimeScript RT reagent Kit (Promega, Madison, WI, USA). A stem-loop quantitative RT-PCR was carried out to detect the expression of mature miR-182 using ABI TaqMan® MicroRNA Assay kit (Applied Biosystems, Foster City, USA) and gene-specific primers (ABI) using an ABI 7500 Real-Time PCR system. mRNA levels of SATB2 gene were measured as previously described [18,21].

### Vector preparation

To generate pLV-miR-182 expression vector, a 110-bp DNA fragment corresponding to pre-miR-182 was amplified and cloned into pLVTHM lentiviral vector. pLVTHM lentiviral vector encodes enhanced green fluorescent protein (EGFP) which has been optimized for brighter fluorescence and greater expression in mammalian cells. The previously described vector pCAG-SATB2 [18] was used to upregulate SATB2 expression.

A 2771-bp fragment of 3' untranslated region (3’UTR) was amplified in accordance with a previously described procedure [21] to generate pGL3-SATB2-3’UTR. The putative miR-182 binding sites at SATB2 3’UTR were site-directed and mutated using GeneTailor Site-Directed Mutagenesis System (Invitrogen). All plasmids were verified by sequencing.

### Lentivirus production and transduction

Virus particles were harvested 48 hours after transfecting pLV-miR-182 with the envelope plasmid pMD2.G and the packaging vector psPAX2 into 293T cells using lipofectamine 2000 reagent (Invitrogen). The lentivirus generated from the empty pLVTHM vector was used as a control (miR-con). These cells were infected with the recombinant lentivirus- transducing units and 8 mg/ml Polybrene (Sigma, St Louis, Missouri, USA) to generate four stable cell lines: two stable over-expression miR-182 cell line (SW480/miR-182 and DLD-1/miR-182) and two control cell line (SW480/miR-con and DLD-1/miR-con).

### Oligonucleotide transfection

miR-182 mimics and antisense inhibitors containing 2’-OMe (*O*-methyl) modifications were synthesized by GenePharma (Shanghai, China). Oligonucleotide transfection was performed with Lipofectamine 2000 reagent (Invitrogen).

### Luciferase reporter assay

In the presence of either miR-182 or miR-con, the firefly luciferase construct was cotransfected into SW480 cells with a control *Renilla* luciferase vector pRL-CMV (Promega). A dual luciferase assay (Promega) was performed 48 hours after transfection. These experiments were performed independently in triplicate.

### Cell proliferation assay and colony formation assay

Cells were seeded in 96-well plates at 2 × 10^3^ per well. Cell proliferation was evaluated using Cell Counting Kit-8 (CCK-8, Dojindo, Rockville, USA) according to the manufacturer's instructions. For performing colony formation assay, the cells were plated in 6-well plates at 2 × 10^2^ per well and maintained in RPMI1640 containing 10% FBS for 2 weeks. After 2 weeks, the cells were washed twice with PBS, fixed with methanol and stained with 0.5% crystal violet. The number of colonies were counted under a microscope [22].

### Wound healing and invasion assays

Cell migration was assessed by measuring the movement of cells in a scraped, acellular area that was created using a 200 μL pipette tube. The spread of wound closure was observed after 0 and 48 hours, respectively. Photographs were taken to assess the level of migration in each group of transfected cells. Migration was quantified by counting the total number of cells that migrated toward the original wound field. For performing invasion assay, matrigel-coated chambers (BD Biosciences, San José, CA, USA) containing 8 μm pores were used. Cells were seeded into the upper chambers (coated in matrigel) by maintaining a concentration of 2 × 10^5^ in serum-free medium. The lower chamber of the transwell was filled with culture media containing 10% FBS as a chemo-attractant. After the chambers were incubated at 37°C for 48 hours, non-invaded cells present on the top of the transwell were scraped off with a cotton swab. The successfully translocated cells were fixed with 10% formalin. Then, they were stained using 0.1% crystal violet for 30 minutes and counted under a light microscope.

### *In vivo* functions assays

4–6 weeks old Balb/C-nu/nu athymic nude mice were obtained from the Laboratory Animal Centre of Southern Medical University. Mice were housed under pathogen free conditions in a 12 hours dark/light cycle and *ad libitum* access to food and filtered water. For performing tumor growth assay, a total of 2 × 10^6^ cells of SW480 with stable over-expression of miR-182 were injected subcutaneously in upper and lower limbs of right flank of mice, while the control cells were injected into upper and lower limbs of left flank of mice (n = 6 per group). The tumor size was measured using digital calipers every three days. After monitoring for 30 days, mice were sacrificed by cervical dislocation and the tumors were dissected. Tumor volume was calculated as follows: Volume = D × d^2^ ÷ 2, where D meant the longest diameter and d meant the shortest diameter.

For developing the metastatic model, mouse cecum was exteriorized by laparotomy under sodium pentobarbital anesthesia. The subcutaneous tumors were diced into 1 mm^3^ cubes and implanted into the mesentery at the cecum terminus of mice. Then, the gut was returned to the abdominal cavity and closed with surgical drapes. After performing the surgery, the health of these animals was monitored on a daily basis. Body weight of mice was also recorded every three days. Humane endpoints had been planned at the end of experiment and as a means to relive pain or distress. At the end of the experiment (8 weeks), the still alive mice were sacrificed by cervical dislocation. The organs were removed and fixed using 10% neutral buffered formalin. Subsequently, consecutive tissue sections were obtained and stained with haematoxylin-eosin (H&E) to observe the metastatic nodules of organs under microscope. All experimental procedures were performed in strict accordance with the recommendations in the Guide for the Care and Use of Laboratory Animals of the National Institutes of Health. The protocol was approved by the Committee on the Ethics of Animal Experiments of Southern Medical University. All necessary steps were taken to minimize the suffering and distress to the mice.

### Western blot analysis

Protein lysates were separated using 10% SDS-PAGE gel electrophoresis and transferred to PVDF membrane (Amersham Pharmacia Biotech, NJ, USA). The membrane was probed with the following antibodies: anti-SATB2 (Abcam, Cambridge, UK), anti-Snail (Abcam, Cambridge, UK), anti-E-cadherin (Cell Signaling Technology, Inc), and anti-Vimentin (Cell Signaling Technology). Finally, the membrane was probed with HRP (horseradish peroxidase)-labeled goat-antimouse IgG (Santa Cruz Biotechnology, USA) and detected by chemiluminescence. A polyclonal anti-*β*-actin or anti-Tublin antibody was used as a protein-loading control. The intensity of protein fragments was quantified using the Quantity One software (4.5.0 basic, Bio-Rad).

### Statistical analysis

All statistical analyses were performed using the SPSS 16.0 statistical software package. In at least three independent experiments, the data were presented in terms of mean ± SD. The differences between variables were assessed by the following three statistical tests: χ^2^ test, Fisher’s exact test, or One-way ANOVA. A *p* value less than 0.05 was considered to be statistically significant.

## Results

### Elevation of miR-182 expression in CRC tissues and cell lines

**Figure 1:**
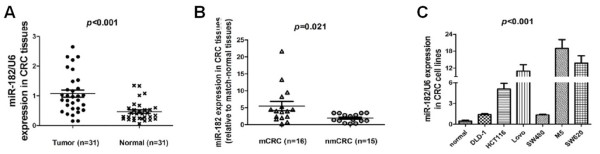
**The expression of miR-182 was up-regulated in CRC cells and tissue specimens using quantitative real-time polymerase chain reaction (qRT-PCR). (A)** The expression levels of mature miR-182 in paired CRC and adjacent normal tissues. **(B)** The miR-182 expression in CRC tissues with or without metastases relative to match-normal tissues. nmCRC denotes CRC tissues without metastases; mCRC denotes CRC tissues with lymph node metastases. **(C)** The relative expression of miR-182 in CRC cells and normal tissues samples. miRNA abundance was normalized to U6 RNA.

We first assessed the expression level of miR-182 by a stem-loop quantitative RT-PCR in 6 CRC cell lines, 31 CRC tissues and paired non-cancerous tissues. The results indicated that miR-182 was markedly up-regulated in CRC tissues compared with adjacent non-cancerous tissues (*p* < 0.001, Figure 1A). We found that the up-regulation of miR-182 in tumor samples was associated with lymph-node metastasis to a significant extent (*p* = 0.021, Figure 1B). However, it was not associated with tumor differentiation degree to a significant extent (*p* > 0.05). In all six CRC cell lines, miR-182 was significantly higher than in all of the non-cancerous tissue samples (*p* < 0.001, Figure 1C). Among CRC cell lines or subclones, miR-182 was lower expression in the cells that originated from the primary tumors (SW480 and DLD-1) than those that originated from metastatic foci (SW620, Lovo, M5 and SCP51). This association indicated that miR-182 might well have a key role in CRC metastasis.

### miR-182 accelerated CRC cells growth, invasion, and migration *in vitro*

**Figure 2:**
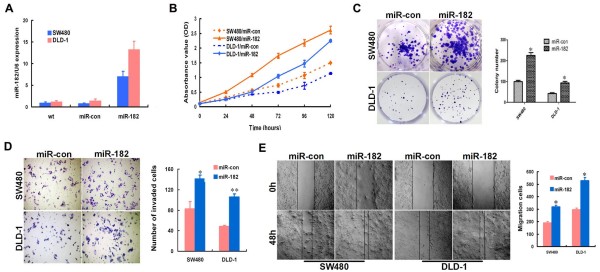
**Ectopic over-expression of miR-182 promoted aggressive phenotypes of CRC cells *in vitro*. (A)** Real-time PCR analysis of miR-182 expression level in SW480 and DLD-1 cells after ectopic over-expression of miR-182. **(B)** Effect of miR-182 on cell proliferation was measured by CCK-8 assay. **(C)** Comparison of colony formation effect on CRC cell lines. **(D, E)** Effects of miR-182 over-expression on cell motilities and invasiveness were determined using matrigel invasion **(D)** and wound-healing **(E)** assays. Data were presented as mean ± SD. The results were reproducible in three independent experiments. * *p* < 0.05, ** *p* <0.001.

To confirm the potential functional role of miR-182 in CRC, we generated two CRC cell lines with stable over-expression and two control cell lines by infecting the recombinant lentivirus. An increased expression of miR-182 upon infection in cell lines was confirmed by real-time PCR (Figure 2A). CCK-8 analysis indicated that the over-expression of miR-182 significantly enhanced the proliferation of cancer cells in both cell lines (*p* < 0.001, Figure 2B). Moreover, compared with the control cells, the ability to form colonies in CRC cells increased owing to a stable over-expression of miR-182 (*p*=0.001 in SW480, *p*=0.003 in DLD-1, Figure 2C). Furthermore, we observed the effect of miR-182 on invasiveness and migration capacity of CRC cells by matrigel invasion assay and wound-healing assay. When matrigel invasion assay was performed on CRC cells and control cells, it was found that the invasiveness was higher for CRC cells with stable over-expression of miR-182 compared with control cells (*p* =0.023 in SW480, *p* <0.001 in DLD-1, Figure 2D). The results also illustrated that exogenetic expression of miR-182 in CRC cells caused a significant increase in cell migratory capacity using a wound-healing assay (*p* =0.002 in SW480, *p* =0.001 in DLD-1, Figure 2E).

### miR-182 promoted tumor growth and metastasis *in vivo*

**Figure 3:**
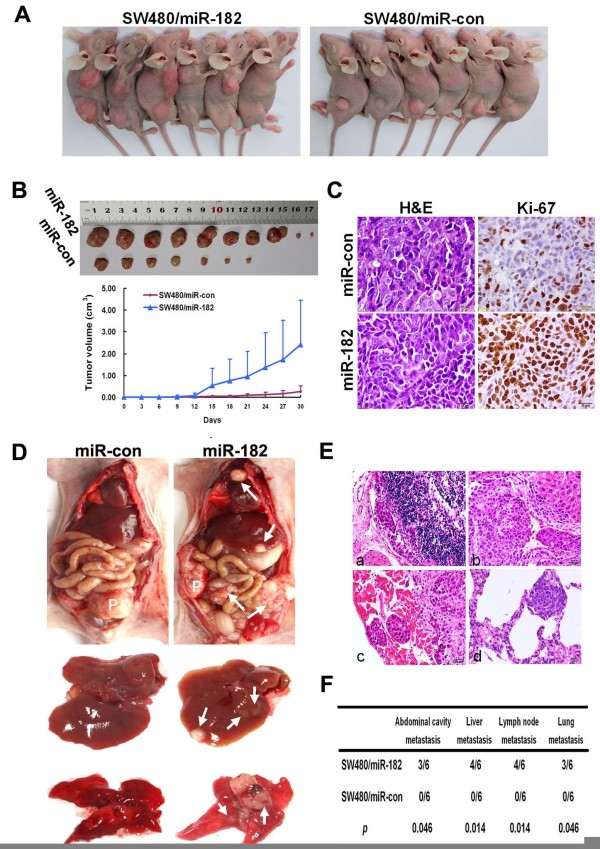
**miR-182 facilitated tumor growth, invasion and metastasis *in vivo*. (A)** SW480 cells were infected with pLV-miR-182 or empty vector virus and injected subcutaneously into nude mice. At 30 days after subcutaneous injection, SW480/miR-182 and SW480/miR-con cells produced primary tumors. **(B)** Representative figure of tumor formed (upper) and growth curve of tumor volumes (lower). Each data point represented the mean ± SD. **(C)** Representative photographs of H&E and immunohistochemical staining for Ki-67 antibody of primary cancer tissues. **(D)** The whole-body images of metastasis in nude mice. P denotes primary tumor. White arrows point to the metastatic nodules. **(E)** Histological images of invasion and metastatic nodules in organs. (a) metastasis of lymph node; (b) metastasis of liver; (c) metastasis of lung; (d) seeding metastasis of abdominal wall. **(F)** Incidence of metastasis in mice that were implanted with SW480/miR-182 or miR-con cells.

The effect of miR-182 on tumor growth was assessed *in vivo* by subcutaneously injecting of SW480/miR-182 and SW480/miR-con cells into the flank of mice. As shown in Figure 3A and B, the average tumor volume increased in SW480/miR-182 cells than in SW480/miR-con group (*p* < 0.05). IHC staining showed that the Ki-67 index in tumors of miR-182-over-expression group was much higher than that in the control group (Figure 3C).

To evaluate the *in vivo* effect of miR-182 on metastasis of CRC, SW480/miR-182 and SW480/miR-con cells were implanted into the caecum terminus of individuals. After 8 weeks, SW480/miR-182 cells developed dramatic metastatic nodules in liver, lung and peritoneal cavity, whereas SW480/miR-con cells only caused tumor increases without any metastasis (Figure 3D). H&E staining clearly illustrated the tumor invasion in intestinal wall, implantation metastasis of organs in peritoneal cavity, and the metastasis of liver, lymph node, and lung in SW480/miR-182 cells (Figure 3E and F).

### Inhibition of miR-182 reduced the growth, invasion, and migration of CRC cells *in vitro*

**Figure 4:**
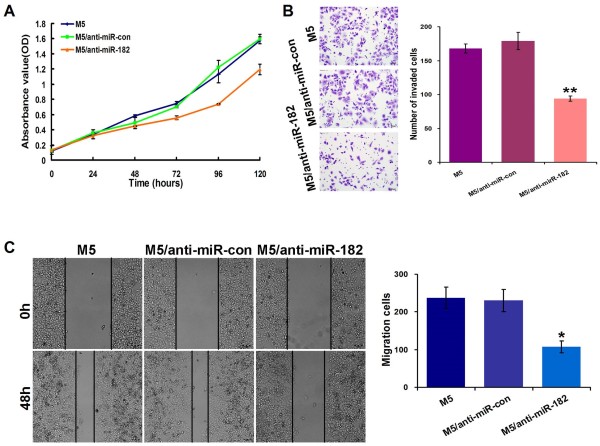
**The down-regulation of miR-182 expression repressed aggressive phenotypes of CRC cells *in vitro*. (A)** Inhibition of miR-182 inhibited cell proliferation of SW620 cells by CCK8 assay. **(B, C)** Inhibition of miR-182 reduced cell invasiveness and migration as determined by matrigel invasion chamber **(B)** and wound healing assays **(C)**. Data were presented as mean ± SD. The results were reproducible in three independent experiments. * *p* < 0.05, ** *p* <0.001.

To confirm the effects of miR-182 on modulating the malignant phenotypes of CRC cells, we also investigated the change of aggressive phenotypes of CRC cells after reducing expression of miR-182. miR-182-specific inhibitor transfection was employed to inhibit miR-182 expression in M5 cells, which had high endogenous miR-182 expression. As shown in Figure 4A, a significantly slower proliferation rate was observed in miR-182 inhibitor-transfected cells than in the control cells (*p* < 0.001). Moreover, the matrigel invasion and wound-healing assays confirmed that the inhibition of miR-182 expression reduced the invasiveness and migration of M5 cells, compared with the control cells (*p* < 0.001 and *p* = 0.001, Figure 4B and Figure 4C).

### SATB2 was a direct target of miR-182 in CRC

**Figure 5:**
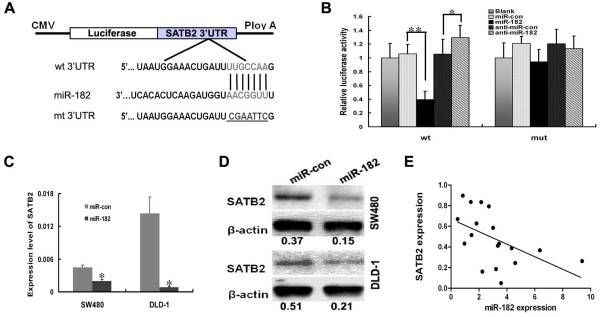
**SATB2 was a direct target of miR-182 in CRC cells. (A)** Schematic illustration of the SATB2 3’UTR- containing reporter constructs. The mutant binding site was underlined. wt denotes wild type; mt denotes mutant. **(B)** Luciferase reporter assays in SW480 cells with cotransfection of wt or mt 3’UTR and miRNA as indicated. **(C, D)** Levels of SATB2 mRNA and protein after miR-182 ectopic over-expression in CRC cells were examined by real-time RT-PCR **(C)** and western blot **(D)**. The immunosignal was quantified using Quantity One Software and relative protein abundance was determined by normalizing with *β*-actin. **(E)** A statistically significant inverse correlation was reported between miR-182 and SATB2 mRNA levels in CRC tissues (Spearman's correlation analysis, *r* = -0.7007; *p* = 0.001, n = 18).

To explore the mechanism that facilitates the effects on proliferation and migration induced by miR-182, we analyzed the putative miR-182 targets by a bioinformatic screen that was developed using three algorithms: TargetScan, PicTar, and miRanda. We focused on SATB2 from which orginated 3’UTR containing a binding site of miR-182. The 3’ UTR sequence of SATB2 (wt 3’UTR) or the mutant sequence (mt 3’UTR) was cloned into a luciferase reporter vector (Figure 5A). A luciferase reporter assay was carried out to determine whether miR-182 can directly regulate the expression of SATB2 in CRC SW480 cells. The results indicated a significant decrease in luciferase activity that was induced by miR-182 over-expression when compared with the empty vector control (*p* < 0.001, Figure 5B). Compared with anti-miR-control, the transfection with anti-miR-182 in SW480 cells led to a significant increase in luciferase activity (*p* = 0.009, Figure 5B). However, the activity of mt 3UTR vector was unaffected with a simultaneous transfection with miR-182 (*p* > 0.05, Figure 5B).

### miR-182 expression had a negative correlation with SATB2 expression

We analyzed the changes of SATB2 expression after the ectopic over-expression of miR-182. We found that the over-expression of miR-182 resulted in reproducible and statistically significant reduction of SATB2 mRNA and protein in both SW480 and DLD-1 cells (*p* < 0.05, Figure 5C and D). Furthermore, we compared the correlation between miR-182 and SATB2 expression in clinical CRC tissues. As shown in Figure 5E, a significant inverse correlation was observed between miR-182 and SATB2 mRNA expression (Spearman’s correlation, *r* = -0.7007, *p* = 0.001).

### SATB2 was a tumor-suppressor in CRC and could attenuate miR-182-mediated malignant phenotype in CRC

**Figure 6:**
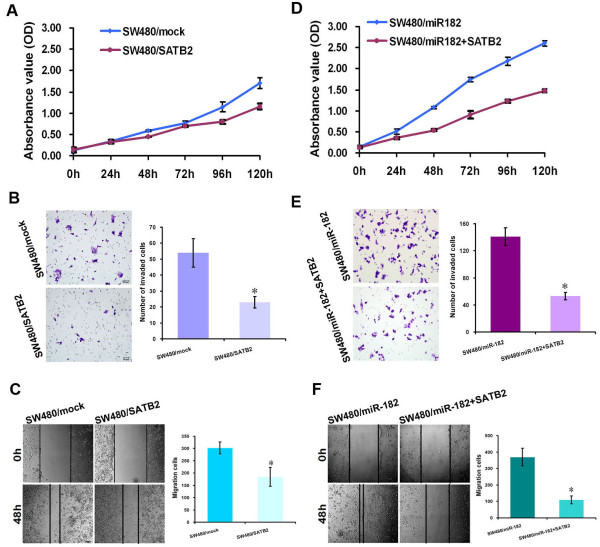
**SATB2 was a tumor-suppressor in CRC and attenuated miR-182-mediated malignant phenotype in CRC. (A)** Cell proliferation was measured by CCK-8 assay to compare the growth difference between SW480 cells with over-expression of STAB2 and SW480 mock cells with transfection of empty vector. **(B, C)** The invasiveness and migration ability were detected by matrigel invasion assay **(B)** and wound-healing assay **(C)** in SW480 cell after SATB2 over-expression. Cell proliferation **(D)**, invasion **(E)** and migration **(F)** changes were detected between SW480/miR-182 cells and SW480/miR-182 cells with over-expression of STAB2. SATB2 could inhibit cell growth, invasion and migration of SW480/miR-182. SW480/miR-182 denoted CRC SW480 cells with stable over-expression of miR-182. * *p* < 0.05, ** *p* <0.001.

The loss of SATB2 mRNA or protein in CRC cells has been observed in our previous studies, but its role in metastasis is yet to be determined. We overexpressed SATB2 in SW480 cells by transiently transfecting with pCAG-SATB2 vector. Compared with SW480 mock cells that were transfected with empty vector, the ectopic over-expression of SATB2 induced obvious repressions of cell proliferation, cell mobility and invasion (*p* < 0.05, Figure 6A-C).

To determine whether SATB2 repression involves the effects of miR-182-mediated cell behavior, we performed rescue experiments by transfecting pCAG-SATB2 vector into CRC cells which had an over-expression of miR-182. Indeed, SATB2 restoration in SW480/miR-182 cells could inhibit both cell proliferation and migration ability compared with that of SW480/miR-182. It could also attenuate the effects of miR-182 in CRC cells (*p* < 0.05, Figure 6D-F).

### Overexpression of miR-182 induces CRC cells metastasis though EMT pathway

**Figure 7:**
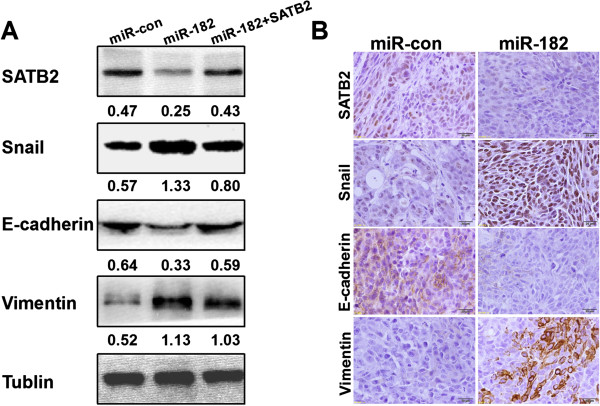
**Over**-**expression of miR-182 induced epithelial mesenchymal transition *in vitro *and *in vivo*. (A)** The expression of epithelial markers, mesenchymal marker, Snail, and SATB2 were compared by western blot analysis in SW480/miR-con, SW480/miR-182 and SW480/miR-182 with exogenetic overexpression of SATB2. Tublin was used as a loading control. **(B)** Immunohistochemistry staining of indicated proteins of the tumors in caecum terminus of nude mice.

We assessed the epithelial and mesenchymal markers with western blot technique. As expected, the expression levels of Snail and mesenchymal maker Vimentin were strikingly upregulated in miR-182-overexpression cells, whereas SATB2 and epithelial marker E-cadherin levels were downregulated. Thereafter, SATB2 overexpression could significantly attenuate the expression changes of the above markers that are associated with miR-182 (Figure 7A).

Furthermore, IHC staining illustrated that the tumors in caecum terminus of mice that originated from SW480/miR-182 cells had increased expression of Snail and Vimentin. Moreover, these tumors that originated from SW480/miR-182 cells had decreased expression of SATB2 and E-cadherin, compared with that from SW480/miR-con (Figure 7B). These data indicated that the over-expression of miR-182 induced the EMT of SW480 cells both *in vitro* and *in vivo*.

## Discussion

miR-182 belongs to the miR-183 family which is comprised of miR-96, miR-182 and miR-183. They are coordinately expressed from a single genetic locus that is located at human chromosome 7q32.2. In recent times, miR-182 has emerged as an important regulator of various physiological and pathological processes [8,11,23]. However, the functional analyses of miR-182 in tumorigenesis have yielded inconsistent results in different cancer models [7,9,12]. In this study, we extend the study of miR-182 in tumor. We observed that the upregulation of miR-182 frequently occursred in CRC tissues, and that miR-182 high-expressed in CRC patients with metastases compared with those of patients without metastasis. In functional studies, overexpression of miR-182 dramatically promoted CRC cells proliferation, invasion and migration *in vitro* and *in vivo*. Moreover, miR-182 could suppress SATB2 via directly targeting its 3’-UTRs, and subsequently regulate EMT signaling. Therefore, these results firmly illustrated that miR-182 acts as a pivotal mediator in the development and progression of CRC.

In the previous study, it was revealed that miR-182 plays an oncogenic role in breast cancer [13]. It was shown that miR-182 is associated with poor survival of breast cancer patients and promotes breast cancer metastasis [12]. In melanoma, an over-expression of miR-182 promoted cell migration, and sustained miR-182 suppression that prevented liver metastasis of melanoma cell in mice [14,15]. On the other hand, miR-182 suppresses tumorigenesis of lung adenocarcinoma [7]. A recent systematic meta-analysis revealed that miR-182 is deregulated in colon or colorectal cancer [24]. The up-regulation of miR-182 in CRC was determined by miRNA microarray or quantitative RT-PCR analysis was consistent across three studies [16,17,25]. Our results indicate that miR-182 was remarkably up-regulated in CRC tissues compared with adjacent non-neoplastic tissues. This is consistent with the previous findings of CRC studies [16,17,25], suggesting that a positive role for miR-182 in tumorigenesis.

To reveal the unambiguous role of miR-182 in CRC cells, we established two stable miR-182-overexpression cell lines to test the effect of miR-182 on the behaviors of tumor cells. We conducted our search for determining the effects of miR-182 on CRC cells growth. The results indicated that miR-182 promoted cell growth and colony formation and accelerated tumorigenesis in murine model of CRC xenograft. Thus, the confirming results helped us in deducing that miR-182 is an oncogenic miRNA for CRC. In this context, we also observed that the expression level of miR-182 in patients with lymph node metastases was higher than that in patients without metastases. The expression level of miR-182 in the metastasizing cells was higher than with little metastatic powers. This indicated that the up-regulation of miR-182 can be considered as a predicted factor of metastasis for CRC patients. Indeed, the functional assays *in vitro* revealed that miR-182 can facilitate tumor invasion and migration. In animal models, the over-expression of miR-182 was sufficient to promote the metastasis of CRC cells to lung, liver, lymph node and organs in the peritoneal cavity. This correlation indicates that miR-182 might be playing a pivotal role in CRC metastasis.

In general, each miRNA regulates hundreds of target genes, influencing on almost every biological pathway. A number of mRNAs involve in distinct pathways are targeted by miR-182. In bladder cancer, miR-182 promotes the cell proliferation, migration and invasion by suppressing Smad4 and RECK [26]. miR-182 can target tumor suppressor gene FOXO1 and functions as oncogene in endometrial cancer and breast cancer [13,27]. miR-182 also suppresses cylindromatosis (CYLD) to sustain NF-κB activation in glioma [28]. A recent study revealed that miR-182 represses the expression of MIM (missing in metastasis gene) and promotes breast cancer metastasis [12]. These results indicate that miR-182 exerts its own functions depending on its specific target genes. To further explore the mechanism through which miR-182 regulates CRC invasion and metastasis, we analyzed miR-182 targets. Among these predicted targets, SATB2 was of particular interest as our previous studies have reported the correlation of its silencing with CRC metastasis. 3’UTR of SATB2 mRNA contains a complementary site for the binding region of miR-182. We conducted luciferase activity assays to validate the targeting of SATB2 by miR-182. The activities of SATB2 3’UTR luciferase reporter were responsive to miR-182 over-expression. Endogenous SATB2 expression in both mRNA and protein decreased in miR-182 over-expression CRC cells. In this study, we have clearly illustrated that miR-182 might directly regulate SATB2 expression by inducing mRNA degradation and translational suppression.

SATB2 is a member of nuclear matrix-attachment proteins family that recognizes AT-rich DNA sequences at the base of looped-out chromatin. SATB2 is involved in various important genetic processes, such as chromatin condensation, interaction with other chromatin remodeling complexes, and regulation of transcriptionc [29-31]. SATB1 is found to be a switch to control tumor growth and metastasis of breast cancer [32] and CRC [33]. In previous studies, we observed that the expression of SATB2 mRNA and protein was lower in metastatic CRC tissues and cell lines [18]. Furthermore, we and other group have demonstrated that the down-regulated expression of SATB2 is associated with metastasis and poor prognosis in CRC [18,34]. SATB2 is a target gene of miR-31 [21]. However, the function of SATB2 has never been documented in CRC. Here, we assessed the functions of SATB2 in CRC cells growth and metastasis with an approach of over-expression. The results indicated that SATB2 suppressed proliferation, migration and invasion of CRC cells. Furthermore, the most important effect exerted by miR-182 on cell proliferation, invasion, and migration is partially reversed after transfecting with a SATB2 expression vector. Both miR-182 and SATB2 affect migration and invasion of CRC cells but in an opposite direction. These findings indicate that SATB2 might be an important target of miR-182, wherein it regulated invasion and metastasis in CRC.

In addition, our study revealed the functional mechanism of miR-182 in CRC metastasis. Epithelial-mesenchymal transition (EMT) appears to be a key event in tumor invasion and metastasis. Epithelial cells lose their epithelial adherence and tight junction proteins, lose their polarity and cell-cell contact, and undergo remarkable remodeling of the cytoskeleton resulted in facilitating cell motility and invasion. The reintroduction of miR-182 into CRC cells induced EMT, indicating the down-expression of epithelial markers and up-expression of the mesenchymal markers. However, Qu et al. illustrated the miR-182 induced mesenchymal to epithelial transition (MET) via repressing SNAIL2 in prostate cells [35]. These controversial results indicate that the role of miR-182 is possibly tumor-specific and highly dependent on its targets in different cancer cells. Indeed, the tissue- and time-dependent expression of miRNAs influences protein translation during distinct cellular processes. The aberrant expression of their target genes affected different biological pathways having diverse function [36]. One of the limitations of our study was that it did not exhibit the direct evidence of EMT-related molecules that were regulated by miR-182/SATB2 in our studies. It was demonstrated that SATB2 forms a complex with histone deacetylase 1 (HDAC 1) *in vitro* and *in vivo*. SATB2/HDAC1 complex can regulate gene expression through recruiting chromatin remodelers [31]. Furthermore, histone deacetylase inhibitor treatment enhances Snail acetylation and induces epithelial-mesenchymal transition via inducing Snail transcription [37]. Here, we put forward a hypothesis that SATB2 might recruit HDAC1 to remodel chromatin of Snail, and further regulate the expression of Snail, a key factor in EMT, which needs to be confirmed in further studies.

## Conclusions

In this study, we demonstrated that miR-182 acts as a tumor growth- and metastasis-promoter in human CRC through repression of SATB2. miR-182 inhibitor can be considered as a novel therapeutic target for CRC patients, especially metastatic CRC.

## Abbreviations

miRNA: microRNA; CRC: Colorectal carcinoma; SATB2: Special AT-rich sequence-binding protein 2; EMT: Epithelial-mesenchymal transition; UTR: Untranslated region; FBS: Fetal bovine serum; qRT-PCR: Quantitative RT-PCR; wt: Wild type; EGFP: Enhanced green fluorescent protein; CCK-8: Cell counting Kit-8; H&E: Haematoxylin-eosin.

## Competing interests

The authors declared that they do not have any competing interests.

## Authors’ contributions

SW designed research. MY, JY, DJ and WL contributed in the experimental procedures. MY and SW analyzed the data. SW and YD supervised all the work. All the authors have read and approved the final manuscript.
